# A social media network analysis of trypophobia communication

**DOI:** 10.1038/s41598-022-25301-3

**Published:** 2022-12-07

**Authors:** Xanat Vargas Meza, Shinichi Koyama

**Affiliations:** 1 Faculty of Library, Information and Media Sciences, University of Tsukuba, Tsukuba, Ibaraki Japan; 2 Present Address: Global Innovation Research Organization, Ritsumeikan University, Ibaraki, Osaka Japan; 3 Faculty of Art and Design, University of Tsukuba, Tsukuba, Ibaraki Japan

**Keywords:** Psychology, Risk factors

## Abstract

Trypophobia has attracted scientific attention in recent years. Few related studies have recruited participants using online methods, and even less is known about health communication in an environment where trypophobia was first widely discussed (i.e., the Internet). This study describes communication patterns in a Facebook group for trypophobia by detecting frequent topics, top contributors, and their discourses. We identified key commenters and performed word frequency analysis, word co-occurrence analysis, topic modeling, and content analysis. Impactful users posted and replied more often when discussing peer-reviewed science. Triggering content was actively removed by the group administrators. A wide variety of triggers not discussed in trypophobia-related literature were frequently mentioned. However, there was a lack of discussion on peer-reviewed treatments. The combination of a few expert and many supportive amateur gatekeepers willing to understand trypophobia, along with active monitoring by administrators, might contribute to in-group trust and the sharing of peer-reviewed science by top users of the trypophobia Facebook group.

## Introduction

Visual discomfort associated with clusters of holes has attracted scientific attention after becoming a widely discussed topic on the Internet. The term *trypophobia* was apparently coined by an unidentified Irish woman on a Web forum in 2005^1^. Thereafter, it was initially only used in niche communities. The Google search volume for trypophobia remained flat throughout the 2000s and did not reach a peak until December 2015^2^.

This was the year in which the first trypophobia questionnaire^3^ was published. Media outlets such as *Business Insider* and *Buzzfeed* posted articles on the matter by the end of that year, while Facebook and Reddit groups, Internet domains, memes, and YouTube videos multiplied. Internet forums facilitate communication among groups with specific health issues. However, at the same time, search engines favor extreme content, prompting the sharing of visual images that might cause trypophobic symptoms. Therefore, an empirical study of communication patterns related to trypophobia in online environments is necessary to determine the usefulness of sharing information in such contexts.

### The psychophysiology behind trypophobia

*Visual discomfort* is a term employed by Wilkins to describe adverse events triggered by visual stimuli. It is common in individuals suffering from migraine and epilepsy^4^. The first comprehensive literature review on this phenomenon was compiled by Aminuddin and Lofti^5^. However, trypophobia has been frequently studied based on visual perception.

Studies of trypophobia in children include what could be considered the first scientific account of the fear of holes^6^. Visual discomfort may be an instinctive, rather than a learned, response to distinctive features of visual stimuli^7^. Cognitive-behavioral therapy (CBT) and sertraline have both been found to be effective treatments for children and teenagers^8–11^.

Most studies on trypophobia, however, have involved adult participants. A self-report^12^ revealed the success of art therapy as a medium of expression, but not a cure, of the phobia. Another case report^13^ found an association between the medicament gabapentin and fear of clusters of holes. Cole and Wilkins^14^ called this fear *trypophobia* in their academic work, in which they proposed a rating scale that was later helpful for noticing the increased heart rate, heart rate variability, and larger hemodynamic response in posterior cortical areas among trypophobics exposed to uncomfortable images^15^. The validity of the trypophobia questionnaire has been assessed in other studies^16,17^, and an image dataset to predict visual disgust has been developed^18^.

Trypophobic discomfort can be caused by mid- and low-frequency visual components^19^, and disgust can be determined by cognitive appraisal^20^. There are indications that trypophobic disgust is affected by background images^21^, trypophobic image gists capture visual attention^22^, natural stimuli may cause more discomfort than man-made ones, and populations of different regions may have different levels of trypophobic tendencies^23,24^. This is relevant considering most studies on trypophobia have been published by scholars in Western countries. Visually discomforting images evoke augmented early posterior negativity^25^, and the emotional information of such images is processed unconsciously^26^. Furthermore, the patterns of small clusters of objects may play a much larger role than the amplitude spectrum in determining visual comfort^27^.

Studies that recruited participants online^28^ found an indirect effect between social anxiety and discomfort linked to eye and face clusters. Core disgust sensitivity, personal distress, and proneness to visual discomfort are predictors of trypophobia^16^. Moreover, visual discomfort was higher among the participants with a history of skin problems^29^.

Some studies have recruited participants from Facebook trypophobia groups, including Le et al.^3^. A comparison of trypophobics and people with aversion to disease-relevant cluster stimuli found that only the first group presented aversion to clusters without relevance to disease, and this aversion was more associated with disgust^30^. Aditionally, Vlok-Barnard and Stein^31^ described trypophobic symptoms as more common among women and as a chronic, persistent cause of significant distress. In addition, they further confirmed the association with disgust.

In summary, the trypophobia-related literature has largely involved clinical case studies, physiological measurements, and self-reported assessments. The intersection between research on the fear of clusters of holes and Internet-mediated communication has yet to be developed. Therefore, in the following section, we summarize Internet-based and network analysis-based studies with a focus on health communication.

### Health communication in the internet era

Before examining the empirical literature on e-health, it is necessary to address the reliability of digital health records, such as text-based testimonies. This brings us to digital dualism^32^, which involves separating the physical (considered *real*) from the virtual. Haraway^33^ proposed the concept of the cyborg as an entity that is both physical and virtual, with a body extended by technology to function as a communication system. In such an entity, the boundaries of where humanity ends and technology begins are not clear. Therefore, the virtual is one aspect of our identity that facilitates a connection to sources that are otherwise inaccessible and adapt to the context with which they interact.

Another relevant aspect is health literacy, whose definition has matured from individual skills for processing health-related information to include interactions with health systems^34^. It incorporates abilities such as applying health concepts and information to novel situations and activities. This may include participating in ongoing public and private dialogues about health, medicine, scientific knowledge, as well as the cultural beliefs that enable individuals to prevent disease, manage periods of illness, and promote health in public spaces^35^. All these factors are relevant to the communication process facilitated by the Internet environment.

From the aforementioned discourses, we distinguish the need for *trustworthy information* in Internet environments. Some physicians assert that Internet use might exacerbate healthcare costs through nonessential referrals or treatments^36^. There is also evidence that patients can trust misleading information or make health decisions based on sensationalized or emotionally charged content irrelevant to their health context^37^. Furthermore, health anxiety might be related to an increase in online health information searches. This information might result in greater worries among health-anxious individuals if the information stems from a trustworthy website^38^, “trustworthy” being defined as a reputed and official source of information, such as a government or medical authority.

In general, the Internet is not useful for diagnosis^39^; however, there is evidence of high accuracy in forum posts related to specific illnesses^40^. One study found that people tended to take more action based on information from websites than from blogs or personal homepages, with mediation from the perceived level of gatekeeping (information access, curation, and sharing) and information completeness^41^. It seems that individuals prefer to obtain diagnoses of serious conditions from professionals while relying on online emotional support from peers during treatment^42^.

Research in the US estimated that 59% of adults and 86% of youngsters consulted the Web for health information, 64% used health apps, 39% found other people with similar health issues on the Internet, and 20% connected with a health provider online^43,44^. It is also assumed that being younger and having more education are both associated with greater e-health literacy among baby boomers and older adults, while women and the highly educated use social networks more to search for health information^45^.

Regarding social networks, a compilation^46^ of 52 studies applying social network analysis to the development and implementation of behavior-change health interventions found that most studies were descriptive. Only one reported using its results for an intervention. Successful social network interventions mostly occur offline, while online support networks are usually composed of weak relationships that can provide empathetic understanding, validation, role models, and well-being but not instrumental social support or sustainable behavioral change^47^.

Facebook groups have been described as beneficial for health communication in terms of social support for diabetes patients^48^. Moreover, an investigation^49^ of Facebook users’ interactions with status updates according to their feelings found that negative emotions encouraged more comments and supportive language.

The image most often reported to cause trypophobic symptoms is the seed head of the lotus flower^14^, which has been widely shared on the internet. Massanari’s^50^ case study suggests that: (a) viewers who create and share trypophobic images hold dual feelings of attraction and repulsion; (b) trypophobia triggers inspire conversations about intense feelings of revulsion, itching, and nausea; and (c) trypophobia reflects a desire for a collective experience of an affective, sensorial web. This extends web communication from strictly visual to a more embodied and personal experience that affects everyday life. However, this case study did not clearly distinguish between the creators of trypophobic images and those who experienced trypophobic symptoms.

Survey questions related to antisocial attitudes can generate inaccurate estimates owing to social desirability bias^51^. Hence, there is a need for alternative methods to analyze behaviors and communication patterns related to trypophobia to complement previous findings. Considering this phenomenon is not widely known, even among physicians and psychologists, social-network-based analysis can provide a more comprehensive picture of the Internet context of trypophobic symptoms.

Because Internet content explaining trypophobia usually includes inciting images, forming a group in which rules against sharing pictures are respected may make participants feel comfortable communicating aspects of their daily experiences without worrying about triggers. If downplaying the importance of online communication can worsen health conditions, reinforcing its positive qualities could potentially improve the quality of life of individuals with persistent ailments.

### Research objectives

Because a more comprehensive description of trypophobia-related communication in an online environment is required, the following research questions (RQs) were proposed regarding the related Facebook group:

1. Which type of participants engage in conversations about trypophobia?
2. Which conversation participants have more impact?
3. Which symptoms are discussed frequently?
4. Which triggers are related to the symptoms?
5. Which symptom management techniques are discussed?
6. Which feelings and reactions are caused by the posts?

## Results

### Impactful conversation participants

**Figure 1:**
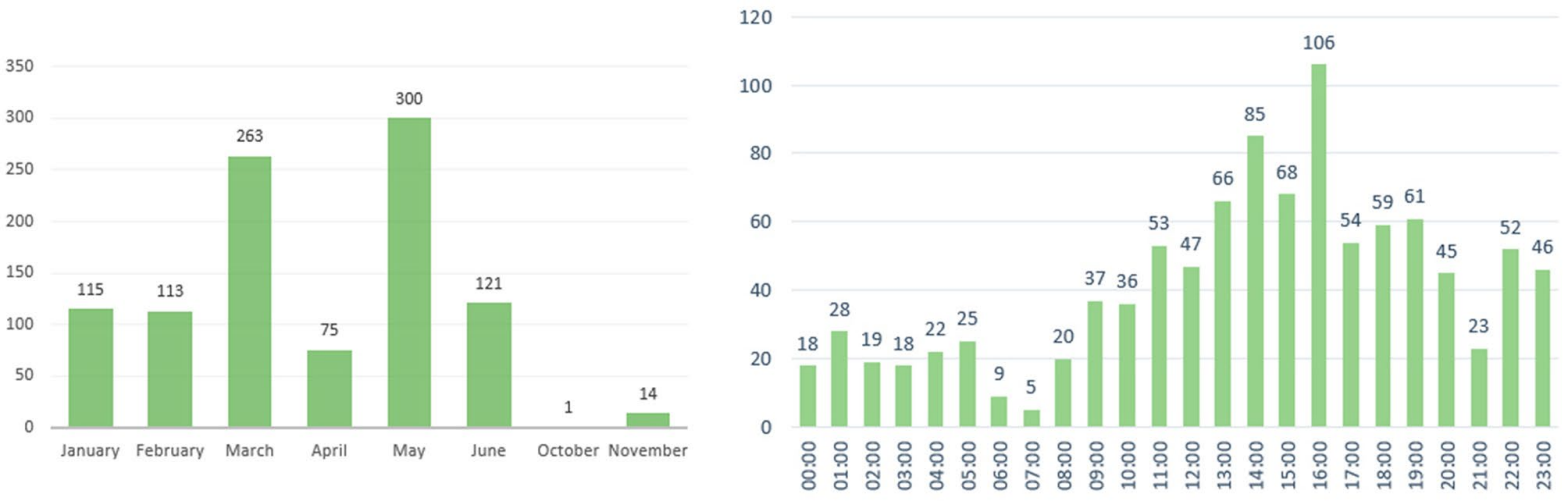
Number of texts posted by Facebook group users per month (left) and hour of the day in UTC-5 (right).

Regarding RQ1, to maintain the confidentiality of the Facebook group users, country data were aggregated per continent (Table 1). A non-parametric Friedman test of the differences between the number of sampled Facebook group members and the number of Internet users in a given country^52^ showed no significant relationship (*χ*^2^ = 35, *p* = 0.24). Google Trends^2^ search volume revealed that the top six countries where the term *trypophobia* has been used since 2004 are all in Southeast Asia, followed by South Africa. Hence, the trypophobia group might be slightly more diverse than expected due to a combination of interest in the aforementioned regions and a higher Internet penetration rate in North American and European countries.A total of 81.89% of the 453 Facebook group sampled users were identified as female, followed by 12.80% identified as male and 5.29% as undisclosed. Users posted 561 texts in March and May, with the day cycle beginning at around 22:00 h. and peaking at 6:00 h. in UTC + 9. Considering that most users with identifiable locations were in the United States and that the American population is mostly concentrated in the eastern region^53^, this hour was transformed to UTC-5. The daily cycle started around 8:00 and peaked at 16:00 (Fig. 1), suggesting that American female users of the Facebook trypophobia group allocated time in the mornings and afternoons for group interaction.

**Table 1:** Number of sampled Facebook group users per continent.

Continent	Number of users	Percent
Africa	19	4.19
Asia	12	2.64
Europe	66	14.56
Latin America and the Caribbean	10	2.20
Middle East	5	1.10
North America	190	41.94
Oceania	12	2.64
Undisclosed	139	30.68
Total	453	100

To answer RQ2, Table 2 shows that the top 10 users in terms of impact posted and replied more often than the average sampled users. Their posts also received more mentions and reactions. Nine of the top users were identified as female, and three disclosed locations in the Middle East, two in Europe, and two in North America. Four talked about triggers, three about science^20^, three about the mitigation of symptoms, and two about experience and severity of symptoms. Four of the top users interacted frequently with other group members. Among these were a group administrator and a new member.

**Table 2:** Top Facebook group users in terms of impact.

User ID	Posts	Comments	Replies	Mentions	Shares	Reactions	Total Impact
57	2	0	1	0	11	30	0.42
343	1	1	8	0	8	59	0.35
304	2	2	19	2	1	57	0.23
446	1	1	6	0	4	22	0.18
452	1	9	8	2	0	49	0.15
189	3	1	5	1	1	23	0.14
387	1	4	3	1	0	80	0.11
317	0	9	9	1	0	22	0.09
433	0	1	4	2	0	7	0.08
179	0	1	3	2	0	2	0.08
Sample average	0.16	1.39	0.63	0.06	0.06	3.54	
Std. dev	0.55	1.36	2.00	0.37	3.76	8.78	
Median	1	1	1	1	1	2	

### Frequent conversation topics and elicited feelings

RQ3–6 were addressed through word frequency analysis and word co-occurrence analysis translated into a graph, topic modeling, and qualitative analysis of the texts. Table 3 shows the topic modeling results with seven main word groups, where five topics (1, 2, 3, 6 and 7) were focused on triggers and symptoms; one (4) was related to coping mechanisms, including art; and one (5) was related to the trypophobia of family members. Among all possible triggers, skin-related ones were in the first topic, the word Surinam (e) was found in Groups 2 and 3, and other natural triggers such as seeds and insects were found in Topic 7. The web address found in Topic 4 is a link to a video claiming to cure trypophobia, but such posts were ignored by group members.

**Table 3:** Topic modeling results.

Topic	Weight	Words
1	10.5418	Holes–feel–trigger–skin–people–trypophobia–makes–images–things–thing
2	1.7855	Stomach–hope–yep–big–scratching–suriname–difference–run–shudder–object
3	1.7282	surinam–part–hole–attacks–photoshopped–blocked–commented–side–design–rice
4	1.7160	lines–start–birth–actual–fascinating–https://loopvideos.com/a–accidentally–art–childhood–paint
5	1.7154	yup–ruin–arms–wanna–clustered–telling–mention–pop–family–irrational
6	1.6704	screen–day–works–bothers–relate–nauseated–cringed–shoes–share–photos
7	1.6661	scare–feelings–kind–thinking–join–organic–quickly–super–wasps–seeds

The settings used to calculate word co-occurrence in Context Software were as follows: 1 mode network with aggregation per corpus, distance 14, and paragraph as the unit of analysis. To avoid triggering effects in trypophobic readers, the resulting network graph is available in Appendix 1. Word size represents frequency, and tie thickness is the strength of the relationship between the words. The term *trigger* was among the top 10 most frequent words, being used 149 times in the sampled texts. It was connected to the following words: *eye*; *skin*; *image*; *picture*; *Google*; *pattern*; *cluster of holes* (*cluster*, *hole*); and *toad*. According to the qualitative analysis, this last term refers to the Surinam toad, found in encyclopedias, museums, television shows, and the Internet. It was considered the first and/or the strongest trigger by several participants of the Facebook group.

The symptoms discussed relate to *body*, *eyes*, *head*, and *skin*. Freaking out (*freak*) and clusters of holes (*cluster, hole*) were connected to *body*, *eye*, and *skin*, whereas *itch* was connected to *body*, *head*, and *skin*. Crawling skin was connected to *eye* and *head; fear*, *gross*, *pattern*, and *feeling sick* to *head* and *skin*; *hate* and *toad* to *head*; and *bother*, *reaction*, and *worse* were connected to *skin*. Moreover, trigger visualization was mentioned even when the eyes were closed. A modularity test performed with Gephi (resolution 0.41, eight-word groups) placed the word *sick* with *pattern*, whereas *freak* was placed with *hole*. This suggests some trypophobic stimuli might elicit more disgust, and others fear. This finding complements previous literature documenting only disgust as relevant.

Regarding the mitigation of symptoms, *destruction* was a word frequently employed in explaining that trypophobics tried to eliminate triggers. They also mentioned closing their eyes: The word *better* was employed in texts that included cleanliness, contact with nature, exposure to triggers, and self-improvement. Therefore, feeling better was mostly related to mitigation of anxiety and stress. Art therapy-related words were found in topic modeling, which suggests they represent common advice. However, there was only one account of successful desensitization in the text sample mentioned by one of the impactful users, which consisted of watching an exhibition by dot artist Yayoi Kusama from a technical and artistic perspective accompanied by close acquaintances.

Although most feelings expressed in the group were connected to symptoms, gratitude to other members was also expressed (the words *thank* and *thanks*). Being understood and sharing experiences are apparently related to such feelings. Furthermore, several frequently used words classified as Kansei (e.g., *because, know, think, understand*) were connected to cognitive processes, pointing to the commenters’ desires to understand trypophobia.

With respect to pronouns, the top word found in the sample was *I*, suggesting that most of the sampled texts were personal accounts. Regarding gender, the pronoun *he* was connected to *eye, skin, bother, picture, thanks, help, please*, and *worse*. *She* was tied to *bad, Google, gross, photo,* and *post*. By revising the comments, we found that while texts describing a man frequently asked for help in mitigating his symptoms, texts describing women listed symptoms and triggers in more detail. This might explain why *gross* was connected to *she* but not to *him*. A more general revision of the comments confirmed that the few account owners who could be identified as male were frequently tied to comments asking and receiving help, hence the great amount of gratitude-related words found in the semantic exploration. Therefore, there were clear differences in terms of the interaction methods used by different genders within the group, even though most members were identified as women.

## Discussion

A study calculated an average of 18 likes per post on American Facebook walls^54^. In comparison, the average of 1.394 likes per post in our sample is modest. This makes sense if trypophobics use the group sporadically to find informational resources or to share updates on their conditions. A sample of students was most active at night^55^, suggesting a more adult demographic in the sampled trypophobic Facebook group.

Previous research suggests female-dominated websites tend to be more supportive^56,57^, as was observed in the trypophobia group. This is in line with the traditional characteristics assigned to women in patriarchal societies, implying that they are allowed to express emotionally charged topics more freely than men; thus, they might feel more comfortable interacting with the group.

The configuration of web platforms impacts who uses them and for what purpose. The open Reddit trypophobia group discussed by Massanari^50^ might be considered as an embodiment of negative aspects of femininity on the Internet since it expresses hate, and even horror, towards ugly, rotten, evil, or corrupted natures, along with a fascination with and attraction to trypophobic images (hence, the sharing of such content). In contrast, the closed Facebook trypophobia group might mostly embody a supportive, protective, and communal femininity while simultaneously being afraid of and/or disgusted by its “dark” side. Therefore, most top users in the sample were identified as women, who, based on Wallace^58^, could be considered as strategic professionals and individual amateurs in terms of information dissemination.

While strategic professionals choose information based on organizational interests or their jobs, individual amateurs are driven more by personal choices^59^. Thus, the user typology has implications for which information tends to be shared. Decentralized gatekeeping, which consists of “micro-level interactions between individuals in a particular collective endeavor” (pg. 357) depends on individual affiliations with reliable information sources, such as news channels and institutions. Social network users usually share non-public affairs and entertainment topics, partly because of their lack of direct access to reliable sources. Hence, the topics contained in trypophobic texts were heavily focused on self-expression and triggers related to mainstream culture. By revising the topic modeling results and cross-checking them with the text, it is clear that art, Internet-based images, movies, and TV commercials were also discussed.

Several university-based researchers interacted in the trypophobia group, and administrators monitored the group for triggering content, removing it at the group users’ requests. One of the most interactive posts in our sample was tied to trigger removal. Therefore, the combination of a few expert gatekeepers with many supportive amateur gatekeepers willing to understand the phenomena, as well as active monitoring by administrators, might contribute to in-group trust and the sharing of peer-reviewed science by top users. Although there were some disinformation and business-focused posts, group members tended to ignore them. This is relevant to understanding the importance of gatekeepers in mitigating fake news on social media platforms, where public health-related disinformation is largely attributed to a white female adult demographic, simply because they are highly active on such platforms.

From the word frequency analysis, we provided more evidence that skin-related symptoms are common and considered serious by trypophobics. Among the triggers, we found further confirmation that natural stimuli might be more triggering than human-made images. This implies that even if trypophobics avoid uncomfortable stimuli on the Internet, they can be exposed to them in their daily lives. For example, Surinam toads were found by group members in both traditional and digital media. Nature and skin-related triggers were frequently mentioned, while a few posts dealt with common foods such as seedy fruits and vegetables, mushrooms, and pasta, or objects such as Christmas lights.

This highlights the need for information on the treatment of trypophobia. However, although a few members recommended therapy, peer-reviewed findings on therapy and medication were largely absent from the frequent words and conversations of top commenters. The Science Alert link did not contain information on treatment either, even though its editorial group comprised journalists who had either a scientific background or who specialized in scientific journalism^60^. As the first treatment-related article was published in 2016, this was a key omission. It should be noted that algorithms for search engines tend to prioritize sensationalist content. As such, they will likely show triggering images or sensationalist texts in the case of the Facebook group, thus discouraging trypophobics from finding the information they require or burying it and making it difficult to find.

Technology should be a tool for normalization, rehabilitation, and treatment in health contexts. In the case of the Facebook trypophobia group, conversations focused on the uniqueness of living with this ailment, emotional support, and the mitigation of symptoms. These are preliminary conditions for greater engagement with experts in diffusing and researching effective treatments for trypophobia. However, the apparatus that serves the consumption of online images can also contribute to visual discomfort. Smartphones and tablets can increase headaches, eyestrain, dry eyes, and sore eyes^61^. As alternatives such as display curvature and task breaks can mitigate discomfort^62^, this is another relevant point for future research on the mitigation of trypophobic symptoms.

Further, analyzing whether there are relationships between trypophobia and different attitudes towards nature and pro-environmental behaviors could help us to better understand why natural stimuli elicit stronger responses among trypophobics. This would bring us closer to developing more effective treatments.

Finally, regarding the emotions elicited by trypophobia images^29^, we confirmed the presence of fear, discomfort, disgust, and nausea, but not positive emotions such as entertainment or enjoyment. Other webometric methods such as sentiment analysis or surveys targeting positive emotions among members of trypophobia-related groups could be employed to clarify this point in future research.

## Conclusions

In the present study, we analyzed communication patterns related to trypophobia. We detected key commenters and performed topic modeling, word frequency analysis, word co-occurrence analysis, and content analysis on a sample of texts from a Facebook group.

The answers to the research questions were as follows:

1. RQ1 and 2: The trypophobia Facebook group was predominantly female.
2. RQ3: Common severe symptoms focused on the skin and were associated with fear and disgust.
3. RQ4: While some triggers were more strongly associated with disgust, others were more strongly associated with fear. Natural-based triggers were considered more serious than man-made ones.
4. RQ5: Symptom management techniques focused on the mitigation of anxiety and stress and the destruction of stimuli. There was a lack of discussion on peer-reviewed treatments, such as cognitive-behavioral therapy or medication.
5. RQ6: While triggers were associated with negative feelings, texts tended to show empathy and support for trypophobics, whereas male commenters frequently used words of gratitude.

Therefore, we can conclude that the usefulness of the Facebook group is limited to support and symptom management techniques, although a high level of in-group trust holds promise for developing further trypophobia research and treatments.

### Limitations

We acknowledge that Facebook data harvesting techniques have been developing rapidly. It would be worthwhile to employ automatic methods with which to collect bigger sized text samples. We also acknowledge a language limitation that could be solved by harvesting data from open posts on Facebook and discussing trypophobia in other languages.

Given that the present study found a wide diversity of triggers not mentioned in previous literature, and given the strong link between triggers and mainstream culture, triggers are likely to change over time. Thus, it would be worthwhile to assess changes in keywords over time as well as the degree of discourse variance among the key commenters, whose importance in conversation dynamics might also be subject to change across time. A larger dataset that would allow user segmentation would be highly advisable for assessing differences in triggers and symptoms.

## Methods

**Figure 2:**
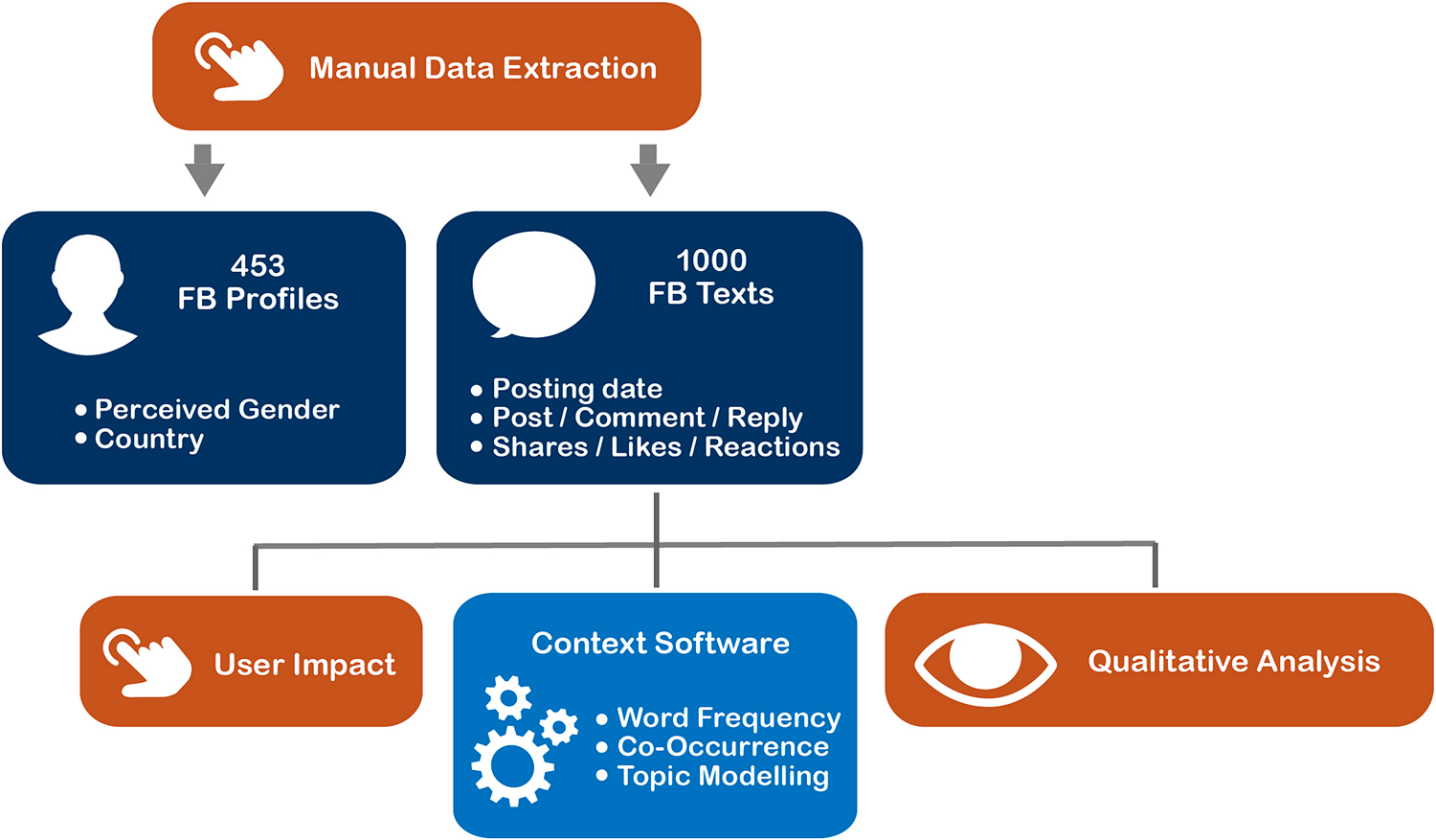
Workflow summary for the present research.

Figure 2 provides an overview of the research procedure. Because Facebook did not allow automated data harvesting in private groups during our research period, we manually extracted 1,000 texts from a trypophobia-related group^63^, with the consent of the moderators and participants, from May to June 2018. This study was considered exempt from ethical review because it was conducted on data from a social network. As such, it did not interfere with any patient or human data beyond measuring Internet activity among Facebook users. This study used profile data from users who consented to Facebook’s public disclosure of their data, indicating that they did not select privacy settings. To protect the individual identities of these users, we anonymized the data.

The collection of texts started at the top of the page and went downwards, with related collected data including posting dates and hours; whether the content was a post, comment to a post, or a reply to a comment; and the number of shares, likes, and reactions. Some authors^64^ note that the Facebook algorithm gives top weight to shares, followed by comments and likes. In the case of our 1,000 texts, most of them (634) were comments. Very few (29) were shares, so we assumed the texts we acquired for this study had high intra-group engagement due to a combination of the Facebook algorithm, which prioritizes sensationalist and visual content, and a high interaction with the texts among the members of the trypophobia group.

The advantage of harvesting the texts by hand is that they could be organized according to the conversations they belonged to, thus making it easier to determine if the group users were self-disclosing their trypophobia or discussing their trypophobia-related symptoms and triggers. Of the 1,000 texts, 700 were of this type. We retained the rest to provide context regarding group dynamics.

To answer RQ1, 453 Facebook profiles associated with the texts were visited to determine whether the user could be visually identified as male or female, and whether their country of origin was public. This user-related information was kept separate from the texts, while both texts and users were assigned alphanumeric identifiers.

To answer RQ2 and part of RQ6, we adapted a measurement of user impact in textual engagement^65^ as follows:

$$ui\, = \,\left( {p/P} \right)\, + \,\left( {c/C} \right)\, + \,\left( {r/R} \right)\, + \,\left( {m/M} \right)\, + \,\left( {s/S} \right)\, + \,(e/E),$$

where user impact *ui* equals user posts *p* divided by total posts *P*, plus user comments *c* divided by total comments *C*, plus user replies *r* divided by total replies *R*, plus mentions of user *m* divided by total mentions *M*, plus user post shares *s* divided by total post shares *S*, plus emotional reactions to user posts *e* divided by total emotional reactions *E*. In our text sample, *P* = 77, *C* = 634, *R* = 289, *M* = 29, *S* = 29, and *E* = 1608. A mention was considered to refer to a user’s name written after the symbol *@.* Emotional reactions consisted of Facebook reactions, such as *Love, Haha, Wow, Sad*, and *Angry*, which were available during the data collection period.

To answer RQ3 to RQ6, we performed word frequency analysis, co-occurrence analysis (pairs of words used together), and topic modeling with ConText software^66^ in the comments. This software was designed to conduct text-based analyses and has been employed in other studies on health communication^67,68^. Most of the 1,000 comments were in English, but Google Translate was applied to 13 comments in French and two in Spanish. Although this software tool tends to be inaccurate for languages other than English, it provides a benchmark that is reliable enough for scientific research^69,70^. Further, the principal investigator was a native Spanish speaker and could ensure that the translations were sufficiently reliable. Words were stemmed, as the text sample was not large. The top terms in frequency were also classified on a scheme based on Vargas Meza and Yamanaka^71^: nouns, Kansei words, verbs, direct objects/topics, place/time-related words, and measures. *Kansei* refers to words related to sensitivity, sense, sensibility, feeling, aesthetics, emotion, affection, and intuition^72^.

Pronouns are frequently omitted from semantic analysis because they are considered as “stop-words.” However, this implies that there are research gaps related to gender that might negatively affect the results of the semantic analysis. As perceived gender influences social expectations^73^, pronouns were also included. Frequent terms were visualized through a graph drawn with Gephi^74,75^. To find topics, ConText employed latent Dirichlet allocation (LDA) based on word co-occurrence. Moreover, to complement the findings, we revisited the 1,000 comments to provide a qualitative (content) analysis.

This study was carried out in accordance with the Declaration of Helsinki and in line with Internet research ethical guidelines (available at https://ahrecs.com/resources/internet-research-ethical-guidelines-3-0-association-of-internet-researchers-aoir-october-2019). The study was approved by the ethical committee of the Faculty of Art and Design, University of Tsukuba, on September 15th, 2017. The approval number was Art and Design 29-11.

## Supplementary Information

Supplementary Information.

## Data Availability

Anonymized data are available through direct contact with the corresponding author.
