# Supplementary material for: A social media network analysis of trypophobia communication
Source: Sci Rep. 2022 Dec 7;12:21163. doi: 10.1038/s41598-022-25301-3 (PMC9729576; doi:10.1038/s41598-022-25301-3)

A Social Media Network Analysis of Trypophobia Communication

Xanat Vargas Meza*, Faculty of Library, Information and Media Sciences, University of Tsukuba, Tsukuba, Ibaraki, Japan; Global Innovation Research Organization, Ritsumeikan University, Ibaraki, Osaka, Japan, kt_designbox@yahoo.com;
Shinichi Koyama, Faculty of Art and Design, University of Tsukuba, Tsukuba, Ibaraki, Japan. skoyama@geijutsu.tsukuba.ac.jp

Appendix 1. Semantic Network of Most Common Words

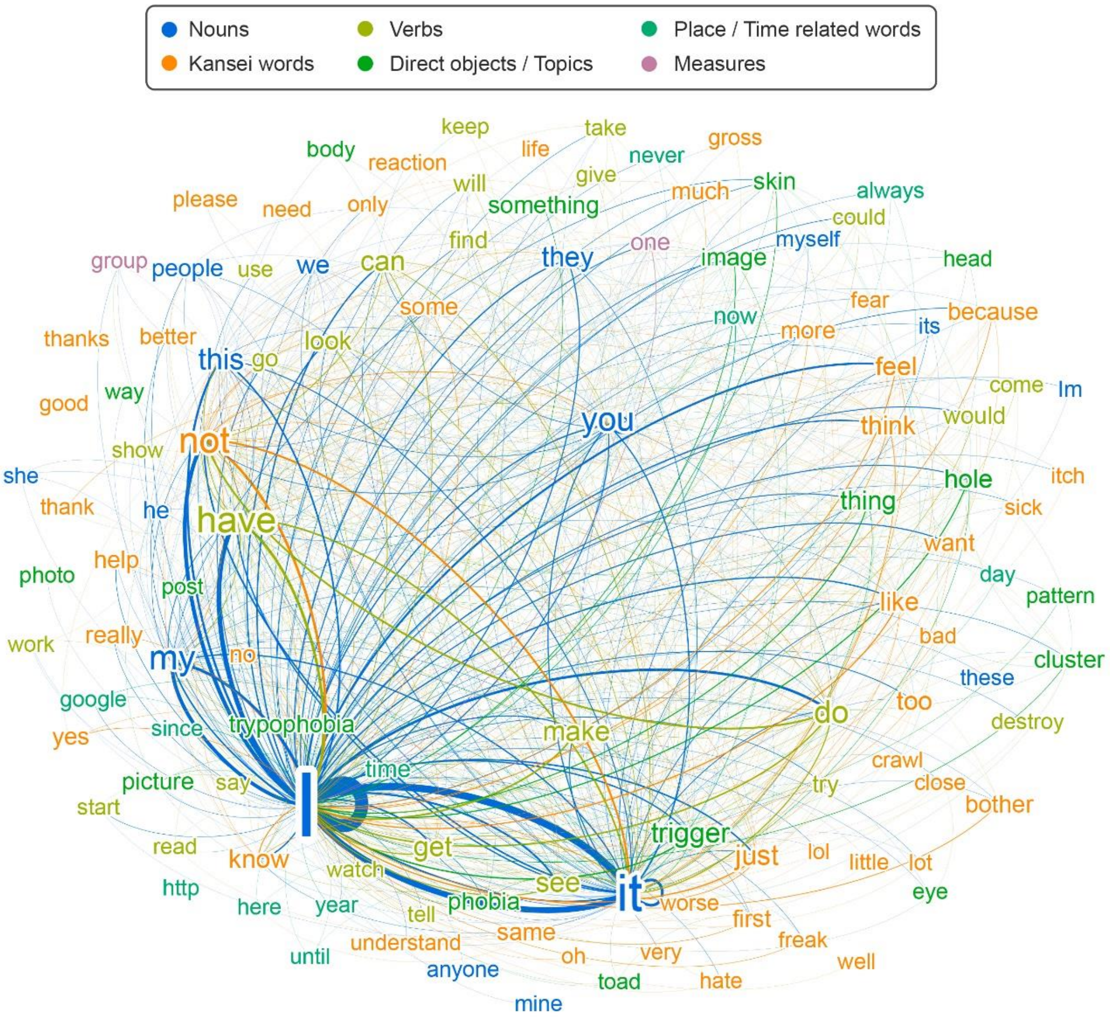

Supplement: Supplementary file 1 — Supplementary Information. [file 41598_2022_25301_MOESM1_ESM.pdf]
